# Immune checkpoint inhibitor-associated diabetic ketoacidosis and insulin-dependent diabetes: a case report

**DOI:** 10.1186/s13256-024-04852-1

**Published:** 2024-12-27

**Authors:** Yungee Jung, Anthony Lau, Joseph Bednarczyk

**Affiliations:** 1 VA Pharmaceutical Sciences, Vancouver General Hospital, 855 W 12th Ave, Vancouver, BC V5Z 1M9 Canada; 2 Emergency Medicine, Vancouver General Hospital, Vancouver, Canada; 3 Faculty of Pharmaceutical Sciences, University of British Columbia, Vancouver, Canada; 4 Faculty of Medicine, Department of Emergency Medicine, University of British Columbia, Vancouver, Canada

**Keywords:** Case report, Diabetic ketoacidosis, Immune checkpoint inhibitor, Immune-related adverse effects, Nivolumab

## Abstract

**Background:**

Immunotherapy, including the use of immune checkpoint inhibitors such as nivolumab, is increasingly common in cancer treatment and can lead to various immune-related adverse effects, including rare cases of diabetic ketoacidosis. This case report highlights an unique instance of nivolumab-induced diabetic ketoacidosis in a patient without prior history of diabetes, emphasizing the importance of careful monitoring even in those without traditional risk factors.

**Case presentation:**

We report a case of a 70-year-old Caucasian male with metastatic esophageal adenocarcinoma who developed diabetic ketoacidosis 3 weeks after stopping nivolumab therapy. The patient had no previous history of diabetes, nor had he used sodium–glucose transport protein 2 inhibitors or corticosteroids. Diagnostic tests confirmed diabetic ketoacidosis, and while he was initially treated following the institutional protocol, he continued to require insulin therapy indefinitely.

**Conclusions:**

This case report underscores the risk of diabetic ketoacidosis linked to nivolumab, even in patients without predisposing factors, emphasizing the need for increased vigilance among both oncologists and physicians. It highlights the importance of monitoring for new-onset diabetes and diabetic ketoacidosis, whether immunotherapy is active or discontinued, and ensuring comprehensive care including hospitalization, insulin management, and diabetes education if diabetic ketoacidosis is diagnosed.

## Background

Immunotherapy is a chemotherapeutic approach used in approximately 15% of patients with various malignancies in a recent population-based study and will consequently be encountered frequently in patients presenting to the emergency department (ED) [1]. Nivolumab, an immunoglobulin monoclonal antibody, specifically targets programmed cell death protein 1 (PD-1) receptors to block pathways cancer cells use to evade immune surveillance by cytotoxic T cells [2]. Immune checkpoint inhibitors (ICI) can lead to various adverse effects due to their impact on the immune system. These include diarrhea/colitis, hepatitis, nephritis, pneumonitis, rash, and endocrinopathies such as, diabetic ketoacidosis (DKA), reported in 0.2–0.9% of cases [2]. While there are currently published case reports and series on ICI-induced DKA, we present a case of nivolumab-induced DKA in a patient with no known prior history of diabetes or other confounding factors. This case is significant as it highlights the potential risk of DKA linked to PD-1 inhibitors and emphasizes the importance of close monitoring, even in patients without traditional risk factors for diabetes.

## Case presentation

A 70-year-old Caucasian male with a history of progressive widely metastatic esophageal adenocarcinoma presented to the emergency department with a 1 week history of dyspnea, nausea, vomiting, and fatigue. He noted significant weight loss of approximately 14 kg over the past few months. He had no history of diabetes mellitus, no prior use of sodium–glucose transport protein 2 (SGLT2) inhibitor or corticosteroids. He also had no family history of diabetes mellitus, endocrinopathies, or autoimmune diseases. The patient’s chemotherapy regimen included nivolumab 230 mg intravenous every 3 weeks, with the last dose administered 21 days prior to presentation. Laboratory findings showed blood glucose of 30 mmol/L, beta-hydroxybutyrate elevated at 9.6 mmol/L, arterial pH 7.2, bicarbonate 13 mEq/L, pCO_2_ 58 mmHg, anion gap 31.4 mEq/L, initial serum potassium 5.3 mEq/L, and hemoglobin A1c 7.5% (previous recent baseline hemoglobin A1c 5.7% when the patient was not on nivolumab therapy). The clinical diagnosis of DKA was made.

The patient was thus treated with the institutional DKA protocol, with an initial bolus of 3 L intravenous crystalloid and an insulin infusion at 0.1 units/kg/hour (equating to 5 units/hour).

By day 2, the patient’s anion gap closed, allowing transition to subcutaneous insulin glargine (15 units daily) and insulin lispro (2 units three times daily with meals). Endocrinology diagnosed new-onset type 1 diabetes mellitus (T1DM) with ongoing high risk of recurrent DKA due to irreversible autoimmune pancreatic beta cell destruction. The diagnosis of nivolumab-induced DKA was made based on the low A1c and the timing of nivolumab administration, though further investigations such as anti-GAD and islet cell antibody testing were not pursued in alignment with the patient’s goals of care. The team recognized the diagnostic challenges, but noted that these investigations would not alter the management and treatment decisions. The patient required insulin glargine 6 units subcutaneous (SC) daily and an insulin sliding scale upon discharge after four days of hospitalization. He received diabetes counseling and insulin teaching from the diabetes nurse educator.

From the patient’s perspective, the team’s prompt diagnosis of ICI-induced DKA and the effective management of the condition significantly improved his symptom relief and quality of life, despite the guarded prognosis. The timely intervention provided critical support and comfort, helping to manage acute distress and offering reassurance during a difficult period. The patient valued the team’s dedication and responsiveness, which made a meaningful difference in his overall experience and care.

## Discussion

This case report adds valuable insight to the existing literature by specifically addressing nivolumab-induced DKA in the absence of other confounding factors, thereby reinforcing the risk of DKA associated with PD-1 inhibitors and highlighting the need for increased vigilance even in patients who are not otherwise predisposed to diabetes. Mae et al. reported cases of delayed immune-related events with nivolumab monotherapy, where T1DM manifested as DKA 4 months after treatment cessation following 12 doses [2]. Similarly, Seo *et al*. described a lung cancer patient who developed new-onset T1DM with DKA 4 months after nivolumab discontinuation following nine treatment cycles [3], requiring ongoing insulin therapy. Capitao *et al*. described a severe DKA case due to immune-mediated T1DM requiring daily insulin, which occurred 25 days after starting nivolumab for stage 4 lung adenocarcinoma. These reports highlight the onset variability from weeks to months following discontinuation of the drug [4]. Conversely, Abdullah *et al*. and Godwin *et al*. reported a rapid onset of insulin-dependent DM and DKA following just two doses of nivolumab [5]. These patients continued to require insulin therapy post discharge from hospital. DKA may also occur from the combination therapy with nivolumab and ipilimumab and with nivolumab and SGLT2 inhibitors.

ICIs may be rechallenged based on clinical judgment of risks and benefits. Some case reports describe successful ICI retreatment while continuing insulin therapy following DKA resolution. However, ICIs may need to be stopped indefinitely especially if immune-related adverse events are severe and life-threatening. In studies of patients retreated with ICIs, the rate of recurrent immune-related adverse events, including DKA, ranges between 18% and 55% [6].

Conversely, some guidelines suggest that the time elapsed since the initiation of ICI therapy is a key factor in determining toxicity [7]. In this case report, the patient began the first cycle of nivolumab therapy three months before presenting with DKA.

The previously published case reports describe investigations with biomarkers that indicate autoimmunity and beta cell destruction. Specifically, they all involved low C-peptide levels, suggestive of insulin deficiency, with or without positive islet-specific autoantibodies [anti-GAD (glutamic acid decarboxylase) antibody, anti-insulin antibody, anti-IA-2 (islet antigen 2) antibody, and anti-ZnT8 (zinc transporter 8) antibody] [2–5]. In this case report, C-peptide and islet-specific autoantibodies were not pursued in the investigations, as their results would not have influenced the management strategy and did not align with the patient's goals of care and values. The decision was made to prioritize interventions directly impacting the patient's immediate clinical needs and preferences. Endocrinology diagnosed this patient with insulin-dependent DKA and diabetes based on T1DM-related symptoms, timing of nivolumab therapy initiation and discontinuation, and immune-related mechanism of action.

**Fig. 1:**
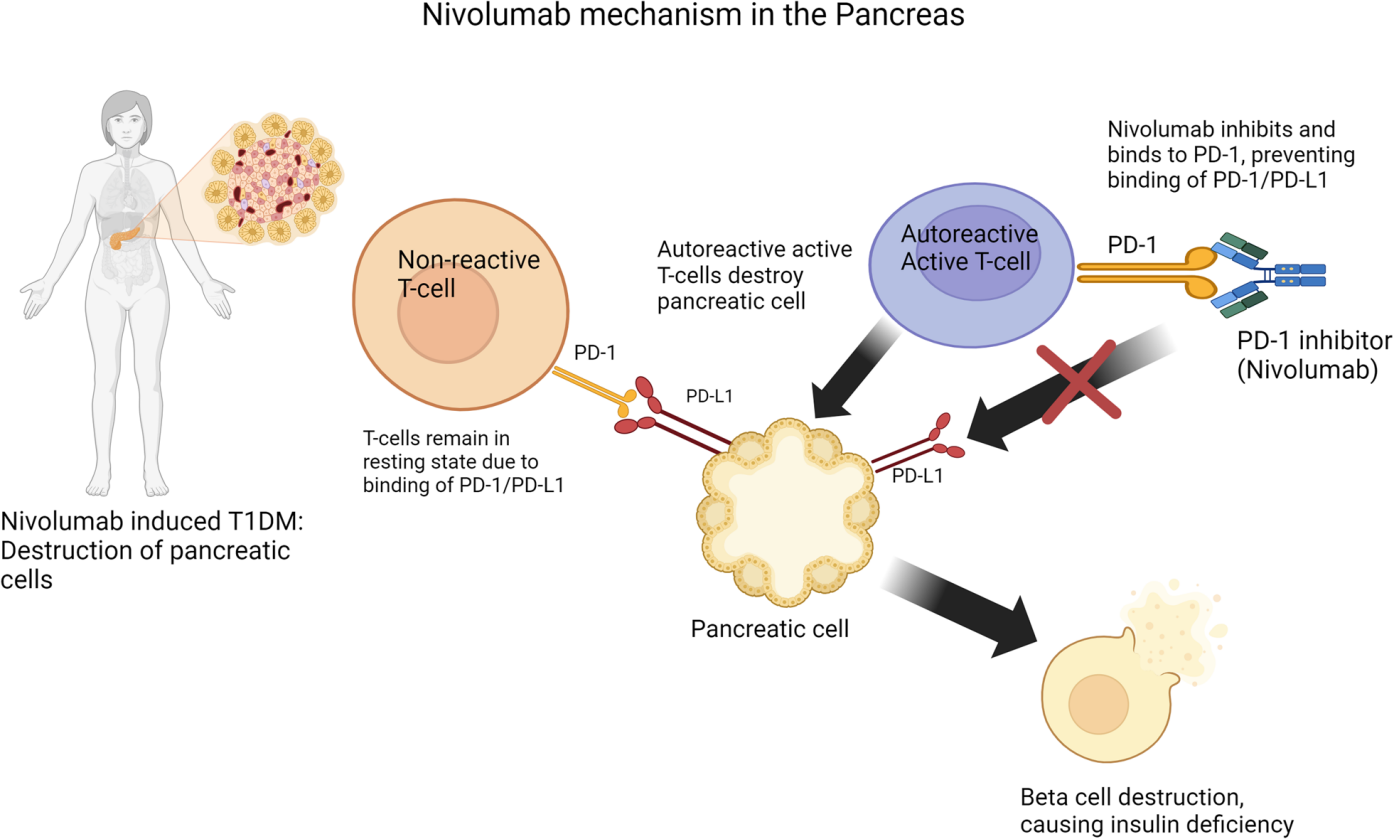
PD-L1 found on pancreatic cells interacts with PD-1 to prevent autoreactive T cell activation from destroying pancreatic cells. Nivolumab inhibits T cell activation in cancer cells by blocking PD-1. This blockade disrupts the resistance of the autoreactive T cells, leading to the destruction of pancreatic cells. Image was created using Biorender

The mechanism of PD-1 inhibitors-induced DM is thought to involve T cell activation. PD-L1, found on pancreatic cells, interacts with PD-1 receptors to prevent autoreactive T cell activation [2]. ICIs such as nivolumab block PD-1, reactivating cytotoxic T cells to inhibit cancer growth. However, this may also induce cytotoxic T-cell destruction of pancreatic beta cells, causing new onset insulin-dependent diabetes mellitus (IDDM) as shown in figure below [3]. This highlights the important point that ICI-induced DKA requires ongoing insulin therapy similar to patients with type 1 diabetes mellitus. Oral antihyperglycemics are not appropriate for managing this permanent condition. In this scenario, IDDM can occur immediately or delayed up to 6 months following nivolumab discontinuation [3] (Fig. 1).

This report describes an unusual presentation of DKA and new onset IDDM occurring 3 weeks after the discontinuation of nivolumab in the absence of other contributory medications or etiologies, which have been described in other case reports as potential confounding factors. Importantly, the patient had no prior DM history, highlighting nivolumab’s potential to induce significant and lasting insulin deficiency. Starvation ketoacidosis was ruled out due to severe hyperglycemia at presentation. Furthermore, acute acidosis from chronic dysphagia was unlikely as the patient showed no signs of refeeding syndrome and had a normal anion gap a few months earlier. According to the Naranjo Algorithm Scale, the correlation between nivolumab and DKA was rated as “probable” [8]. The Naranjo Scale is used to assess the likelihood that a specific drug is responsible for an adverse event, helping clinicians evaluate causality in drug-related side effects.

## Conclusions

Our case report illustrates autoimmune IDDM and DKA can develop in patients without prior history of diabetes who are receiving immunotherapy. Furthermore, DKA can manifest weeks to months after nivolumab therapy cessation. Both oncologists and physicians must remain vigilant for symptoms of new onset DM and DKA, whether immunotherapy is ongoing or has been discontinued. If immunotherapy-associated DKA is diagnosed in hospital, patients require admission for multidisciplinary medical care and stabilization, transition to an outpatient insulin regimen, and diabetes education. Prospective studies are warranted to clarify the incidence, risk factors, and optimal management of nivolumab-induced DKA. By staying alert to the signs and symptoms of DKA in patients who have received nivolumab, oncologists and physicians can play a vital role in timely diagnosis and management of ICI-associated DKA.

## Data Availability

Data may be available from the corresponding author on reasonable request. The authors declare that data supporting the findings of this study are available within the article.

## Abbreviations

DKA: Diabetic ketoacidosis
ICI: Immune checkpoint inhibitor
IDM: Insulin-dependent diabetes
PD-1: Programmed cell death protein 1
T1DM: Type 1 diabetes mellitus
